# Single-cell RNA sequencing uncovers molecular mechanisms of intravenous immunoglobulin plus methylprednisolone in Kawasaki disease: attenuated monocyte-driven inflammation and improved NK cell cytotoxicity

**DOI:** 10.3389/fimmu.2024.1455925

**Published:** 2024-10-25

**Authors:** Minna Yang, Yeshi Chen, Chenhui Feng, Mingming Zhang, Hongmao Wang, Yang Zheng, Xiaohui Li

**Affiliations:** ^1^ Department of Cardiovascular Medicine, Capital Institute of Pediatrics-Peking University Teaching Hospital, Beijing, China; ^2^ Department of Cardiovascular Medicine, Children’s Hospital Capital Institute of Pediatrics, Beijing, China; ^3^ Department of Cardiovascular Medicine, Peking Union Medical College Graduate School, Beijing, China

**Keywords:** Kawasaki disease, methylprednisolone, single-cell RNA transcriptome sequencing, inflammatory response, NK cell cytotoxicity

## Abstract

**Introduction:**

Intravenous immunoglobulin (IVIG) plus methylprednisolone as initial intensive therapy or additional therapy in Kawasaki disease (KD) has been used in clinical practice. However, its molecular and cellular mechanism is unclear.

**Methods:**

We performed single-cell analysis on 14 peripheral blood mononuclear cell (PBMC) samples obtained from 7 KD patients who received either IVIG monotherapy or IVIG plus methylprednisolone therapy. This encompassed 4 samples from KD patients collected before and after IVIG treatment, as well as 3 samples from KD patients before and after IVIG plus methylprednisolone therapy.

**Results:**

Both IVIG monotherapy and IVIG plus methylprednisolone therapy can increase lymphocyte counts (e.g. CD4+T, CD8+T, and gdT cells) to address lymphopenia. They can also decrease monocyte counts and repress the expression of S100A12, NLRP3, and genes associated with immune-cell migration in monocytes. IVIG combined with methylprednisolone downregulates more monocyte-driven inflammatory pathways than IVIG alone. Additionally, this combination uniquely enhances NK cell cytotoxicity by modulating receptor homeostasis, while significantly upregulating interferon-related genes in CD4+ T cells, CD8+ T cells, and B cells, particularly type I interferons.

**Conclusion:**

The combination of IVIG with methylprednisolone attenuated monocyte-driven inflammation and improved NK cell cytotoxicity which might provide clues for pediatricians to consider treatment options for children with KD. Whether the monocyte-driven hyperinflammatory state and NK cell function can be indicators for the clinical choice of IVIG with methylprednisolone therapy in KD needs further investigation.

## 1 Introduction

Kawasaki disease (KD), also known as cutaneous mucocutaneous lymph node syndrome, is an acute vasculitis of childhood that results in coronary artery aneurysms in approximately 25% of untreated cases, which is the leading cause of acquired heart disease in children under the age of five in developed countries (1, 2). The primary therapy of KD including intravenous immunoglobulin (IVIG) and aspirin has already shown proven clinical efficacy (3). However, up to 20% of patients presented unresponsiveness to primary therapy with IVIG and aspirin, placing them at a heightened risk for the emergence of coronary artery abnormalities (CAAs) (4, 5). As the American Heart Association (AHA) recommended in 2017 (2), corticosteroids, one of primary adjunctive therapy, can benefit children at high risk for development of CAAs. Inoue et al. (6) reported a lower incidence of CAAs and retreatment, shorter duration of fever, and more rapid decrease in C- reactive protein (CRP) levels within the steroid-administered cohort. Further, Chen et al. (7) performed a meta-analysis of clinical trials to examine the efficacy of IVIG plus steroid treatment compared to IVIG monotherapy, and concluded that the former strategy is more effective in lowering the risk of CAAs in initial KD treatment.

The underlying mechanisms by which corticosteroids can relieve clinical symptoms of children with KD remain unclear. Saneeymehri S and Wardle A J reported that steroids can help KD patients by downregulating inflammatory mediators, limiting leukocyte migration, and decreasing capillary permeability (8, 9). Chen et al. reported that intravenous methylprednisolone, with its anti-inflammatory functions, suppresses cytokines levels more rapidly than IVIG alone (7, 10). However, there is still a lack of studies systematically evaluating the molecular mechanisms of IVIG plus methylprednisolone in the treatment of KD.

With the use of single-cell RNA sequencing (scRNA-seq), the immune cells and biochemical pathways in KD have become increasingly clear, which can assist in further clarifying the underlying mechanisms of various treatment regimens (11–15). Wang et al. (11) analyze peripheral blood mononuclear cells (PBMCs) from seven KD patients pre- and post-IVIG therapy through scRNA-seq. They reported that after IVIG therapy, a significant decrease in the percentage of CD16^+^ monocytes and an increase in the proportion of plasma B cells were noted, complemented by an upregulation in the expression of genes related to the anti-inflammatory actions of IL10. However, the comprehensive molecular mechanism of IVIG plus methylprednisolone treatment in KD patients is still missing.

In this study, we utilized scRNA-seq to analyze the transcriptomic profiles of 14 PBMC samples obtained from 7 patients diagnosed with KD. In particular, these PBMCs were collected before and after treatment with IVIG or IVIG plus methylprednisolone. Our findings provide valuable insights into the potential mechanisms underlying the therapeutic effects of IVIG plus methylprednisolone and may contribute to the development of novel targeted therapies for KD.

## 2 Methods

### 2.1 Patients

We collected 14 fresh peripheral blood samples from seven patients with acute KD (**Table 1**), recruited from the Children’s Hospital Capital Institute of Pediatrics between February 2022 and March 2022. The patients were diagnosed according to the criteria proposed by the AHA (2). These patients were stratified by the Kobayashi risk score into IVIG non-responders (score ≥ 4) or IVIG responders (score < 4) (16). High-risk IVIG non-responders (score ≥ 4) received an initial dose of intravenous methylprednisolone at 2-4 mg/kg/day. When the fever subsided and CRP levels normalized, the dose of methylprednisolone was tapered within 2 weeks. All patients initially received IVIG at 2 g/kg as a single infusion within 24 hours. The first blood sample was taken from each patient on days 3-7 after the onset of fever before initial therapy. The second sample was collected 72 hours after the initial therapy was completed. With negative PCR and serology results and no COVID-19 positive contact history, all of the subjects were ruled out for SARS-CoV-2 infection. Patients with KD were divided into two different therapy groups: patients treated with IVIG alone (n=4) or IVIG plus methylprednisolone (n=3). The two treatment groups were further subdivided into pre-treatment and post-treatment groups (Pre-Ig, pre-treatment with IVIG only; Post-Ig, post-treatment with IVIG only; Pre-gm, pre-treatment with IVIG plus methylprednisolone; Post-gm, post-treatment with IVIG plus methylprednisolone). All participants were included in the study after obtaining their informed consent. Ethical approval for this study was obtained from the Ethics Committee of the Capital Institute of Pediatrics (SHERLL2021054).

**Table 1:** Demographics of the patient cohorts undergoing single-cell analysis of peripheral blood.

Patient		Diagnosis	Sex	Age (months)	Zmax	Kobayashi Score	Time from KD onset to blood draw prior to therapy(days)	Therapeutic drugs	Response to therapy
1	Pre-gm1	KD	F	55	4.61	6	7	IVIG plus methylprednisolone	Response
	Post-gm1								
2	Pre-gm 2	KD	M	48	1.16	5	5	IVIG plus methylprednisolone	Response
	Post-gm2								
3	Pre-gm3	KD	F	10	1.46	4	4	IVIG plus methylprednisolone	Response
	Post-gm3								
4	Pre-Ig1	KD	M	7	3.04	<4	7	IVIG	Response
	Post-Ig1								
5	Pre-Ig2	KD	M	25	0.63	<4	5	IVIG	Response
	Post-Ig2								
6	Pre-Ig3	KD	F	11	0.82	<4	5	IVIG	Response
	Post-Ig3								
7	Pre-Ig4	KD	M	13	1.76	<4	4	IVIG	Non-response
	Post-Ig4								

KD, Kawasaki disease; IVIG, intravenous immunoglobulin; Pre-gm, pretreatment with IVIG plus methylprednisolone; Post-gm, posttreatment with IVIG plus methylprednisolone; Pre-Ig, pretreatment with IVIG alone; Post-Ig, posttreatment with IVIG alone; F, female; M, male.

### 2.2 Single-cell RNA library preparation and sequencing

For the single-cell RNA library preparation and sequencing, EDTA-vacutainers were used to collect 3 mL of whole blood from each participant of the scRNA-seq. The whole blood samples were immediately transferred to the laboratory on ice. Subsequently, PBMCs were isolated from the whole blood using a centrifuge and a red blood cell lysis buffer provided by Miltenyi Biotec. The viability of PBMCs was assessed using the Countstar Fluorescence Cell Analyzer with AO/PI staining. To further enrich the PBMCs suspension, the MACS dead cell removal kit from Miltenyi Biotec was employed. It was ensured that each sample contained at least 8,000 cells in an appropriate volume of PBMC suspension. Construction of the single-cell libraries was carried out using the 10X Genomics Chromium Controller Instrument, together with the Chromium Single Cell 5’ library & gel bead kit and the V(D)J enrichment kit obtained from 10X Genomics in Pleasanton, CA. The quantification and sizing of the libraries were performed using the Qubit High Sensitivity DNA assay from Thermo Fisher Scientific and the High Sensitivity DNA kit of Bioanalyzer 2200 manufactured by Agilent. All samples were sequenced using Illumina platforms produced by Illumina in San Diego, CA.

### 2.3 Quantification and statistical analysis

#### 2.3.1 Single-cell RNA-seq data analysis

The processing of single-cell RNA-seq data was done as previously described (17, 18). In short, the kallisto/bustools (KB, v0.24.4) pipeline was used to create both raw and filtered gene expression matrices. Then, in Python (v3.8.10), the filtered feature, barcode, and matrix files were examined using the anndata (v0.7.6) and scanpy (v1.7.2) packages. Following the procedure outlined in Wang et al. (19), low-quality cells and potential doublets were filtered, and the gene expression matrix was normalized by library size to 10,000 reads per cell.

The consensus collection of 1500 most highly-variable genes (HVGs) was chosen by prioritizing gene features with high cell-to-cell variation in the data using the sc.pp.highly_variable_genes function, as previously described (17). The methodology for integrating various datasets followed the protocol outlined by *Wang* et al. (19). The data processing pipeline included dimensionality reduction to 30 principal components using PCA, mitigation of batch effects using the Harmony algorithm, and integration of single-cell data with Harmony (20, 21) to ensure fast, accurate, and sensitive analysis. This was complemented by unsupervised clustering utilizing the Louvain algorithm.

#### 2.3.2 Cell clustering and annotations

Two rounds of unsupervised cell clustering were conducted through sc.tl.louvain, using the neighborhood relations of cells. Initially, at a Louvain resolution of 2.0, the analysis discerned eight fundamental cell types, including NK cells, B cells, monocyte cells, dendritic cells, CD4^+^ T cells, CD8^+^ T cells, gamma delta T cells, and megakaryocytes. In the subsequent phase, at the same resolution, it further delineated CD4^+^/CD8^+^ T, B, monocyte, NK, and DCs into subclusters, indicating unique immune cell lineages within the primary categories. Cluster-specific signature genes were identified using the sc.tl.rank_genes_groups function. Each subcluster was manually examined and characterized as distinct on the grounds of expressing at least one signature gene with high expression relative to other cells. Supplemental Figures (**Supplementary Figure S2**) provide canonical marker genes and cluster-specific highly expressed signatures.

#### 2.3.3 Cell state score of cell subtypes

After cluster annotation, specific gene sets were used to compare the physiological activity or overall activation level of cell clusters. The gene sets associated with B cell activation, antigen presentation, S100 protein family, the inflammatory response and immune cell exhaustion were gathered from research that was published (17, 19, 22–26), and the Leukocyte Migration Gene Set (GO:0050900) was gathered from previous reports and the MsigDB database. These 17 cytotoxicity-associated genes (*PRF1*, *IFNG*, *GNLY*, *NKG7*, *GZMA*, *GZMB*, *GZMH*, *GZMK*, *GZMM*, *KLRK1*, *KLRB1*, *KLRD1*, *FCGR3A*, *FGFBP2*, *ZEB2*, *CTSW*, and *CST7*) were used to establish the cytotoxicity score. Cell state scores were computed employing the Scanpy sc.tl.score_genes function, which quantifies these scores by averaging the expression levels of a specified gene set in comparison to a set of reference genes. The Mann-Whitney rank test (two-tail, p<0.01, corrected using the Benjamini-Hochberg procedure) was utilized to statistically test the comparison of the cell state score between two conditions.

#### 2.3.4 PAGA analysis

The developmental path of a cell subclusters was visualized using partition-based graph abstraction (PAGA) within a graph framework. The scanpy functions sc.tl.paga was used to perform PAGA analysis on the neighbor graph obtained during cell clustering, and the sc.pl.paga function was used to draw the PAGA graph.

#### 2.3.5 Identification of Differentially Expressed Genes (DEGs) and functional enrichment

Differential gene expression was analyzed using the sc.tl.rank_genes_groups function in Scanpy, with the Wilcoxon rank-sum test as the default method (test.use=wilcox). The Benjamini–Hochberg method was applied to control the false discovery rate (FDR). DEGs were selected based on a minimum log fold change of 0.5 and an FDR threshold of 0.01. For functional enrichment analysis, the identified DEGs were subjected to Gene Ontology (GO) pathway analysis to elucidate their potential biological functions and the associated signaling pathways

### 2.4 Statistics

All of the statistical analysis, visualization, and methodology mentioned in this paper were carried out using Python. Detailed statistical methods are provided along with the results of the main text, the figure legends, or this section. In all figures, the significant marks ought to be interpreted as follows: ns: p > 0.05; *p ≤ 0.05; **p ≤ 0.01; ***p ≤ 0.001; ****p ≤ 0.0001.

## 3 Results

### 3.1 Single-cell transcriptional profiling of PBMCs from individuals with KD

**Figure 1:**
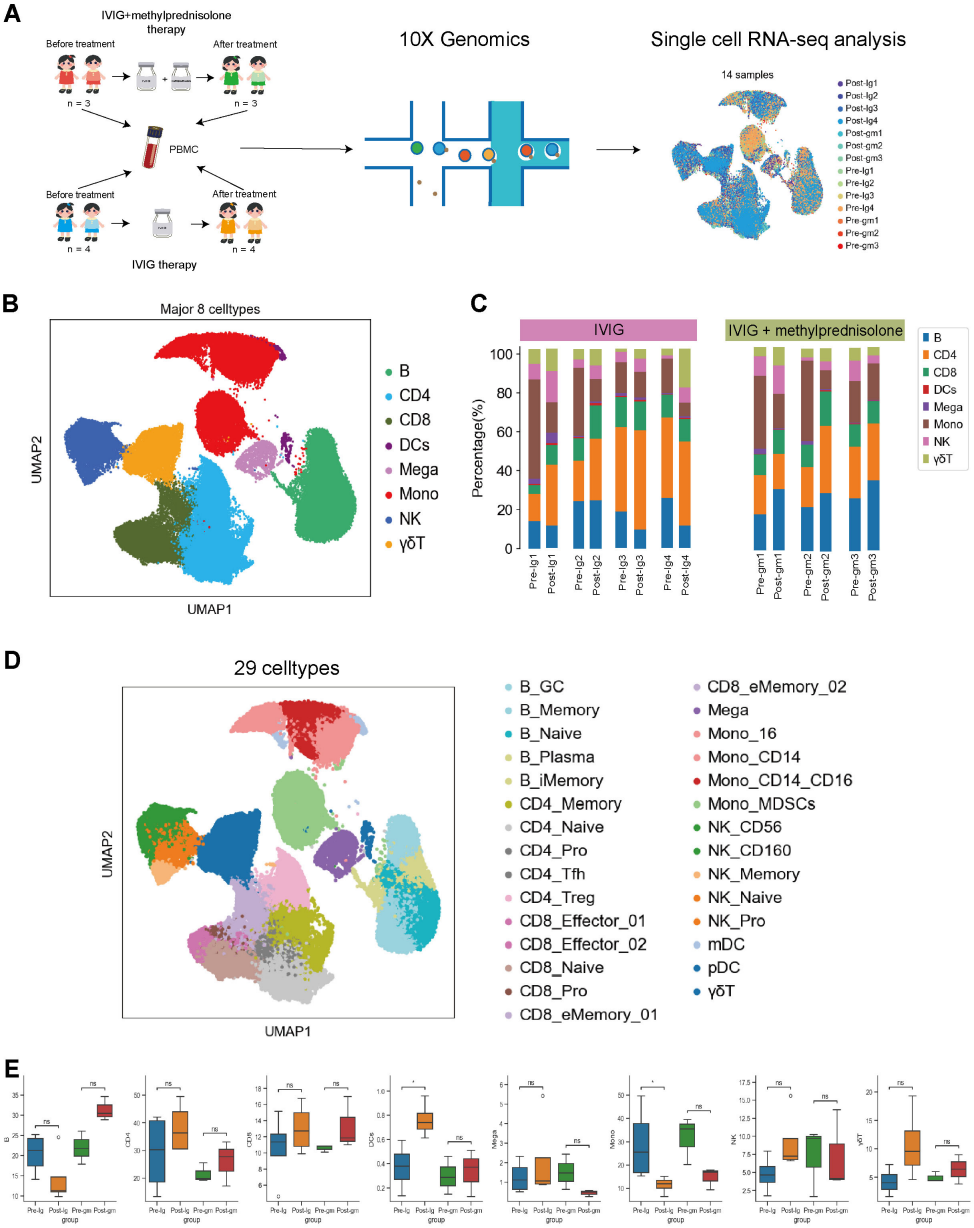
Study design and single-cell atlas of KD patients with different therapies. **(A)** A flowchart of overall study design. Blood samples were collected from 7 patients with KD before and after therapy, among them 3 patients with intravenous immunoglobulin plus methylprednisolone therapy, and 4 patients with IVIG therapy only. **(B)** The results of the first-round cell clustering, which classified all cells in the integrated scRNA-seq dataset into 8 major cell types. **(C)** The proportion of each cell cluster for each individual before and after therapy in each group. **(D)** A two-dimensional visualization of the second-round cell clustering, which classified all cells in the integrated scRNA-seq dataset into 29 distinct cell subtypes **(E)** Comparison of cell amount across patient groups based on scRNA-seq data. Pre-Ig, pretreatment with IVIG only. Post-Ig, posttreatment with IVIG only. Pre-gm, pretreatment with IVIG plus methylprednisolone. Post-gm, posttreatment with IVIG plus methylprednisolone. ns: p > 0.05; *p ≤ 0.05.

A total of 14 PBMC samples were longitudinally collected from 7 patients with KD (**Figure 1A**, **Table 1**). The total number of detected cells passing quality control was 114128, including 29002 cells from the Pre-Ig group, 22413 cells from the Pre-gm group, 32943 cells from the Post-Ig group, and 29770 cells from the Post-gm group (**Supplementary Figure S1**). High-quality cells filtered from different samples were then integrated into an unbatched comparable dataset.

Following clustering with uniform manifold approximation and projection (UMAP), we identified eight major cell types according to canonical marker gene expression, including B cells (CD79A^+^MS4A^+^), CD4^+^ T cells (CD3D^+^CD3E^+^CD40LG^+^), CD8^+^ T cells (CD3D^+^CD3E^+^CD8A^+^), dendritic cells (CST3^+^LYZ^+^), megakaryocytes (CST3^+^PPBP^+^PF4^+^), monocytes (CST3^+^LYZ^+^CD14^+^ or CST3^+^LYZ^+^CD16^+^ or CST3^+^LYZ^+^CD14^+^CD16^+^), natural killer cells (NK; NKG7^+^KLRF1^+^), and γδT cells (CD8A^+^CD8B^+^FCGR3A^+^) (**Figure 1B**, **Supplementary Figure S1C**). We further clustered the major cell groups into distinct subtypes, through which a total of 29 cell subtypes were identified (**Figure 1D**, **Supplementary Figure S2**). Thus, the information-rich dataset can be used to analyze these cell types and subtypes at different resolutions.

### 3.2 The dynamics of major immune cell types in PBMCs

The analysis of major immune cells percentage revealed a remarkable shift in patients with KD when comparing their pre- and post-therapy states. Notably, there was an obvious increase in the majority of lymphocyte populations (e.g., CD4^+^T, CD8^+^ T, NK and γδT cells) and dendritic cells in both treatment groups (**Figure 1E**). This suggests that both IVIG monotherapy and IVIG plus methylprednisolone therapy can effectively address lymphopenia and restore the antigen presentation function during the acute stage of KD. In addition, we observed an increase in B cells following treatment with IVIG plus methylprednisolone, whereas a decrease was noted after IVIG monotherapy. Megakaryocytes increased after IVIG monotherapy but decreased after the combination of IVIG and methylprednisolone (**Figure 1E**). These distinct outcomes suggest that the mechanism underlying the effects of IVIG monotherapy and IVIG plus methylprednisolone therapy might be inherently disparate. Notably, monocytes, an important class of inflammatory cells, showed a significant decline after both treatment strategies (**Figure 1E**). This implies that targeting monocytes could be crucial in mitigating the inflammatory response in KD. Therefore, we further investigated the molecular mechanisms of anti-inflammatory response associated with monocytes within two therapeutic contexts.

### 3.3 IVIG plus methylprednisolone exerted more intense anti-inflammatory effects in monocytes through multiple pathways

**Figure 2:**
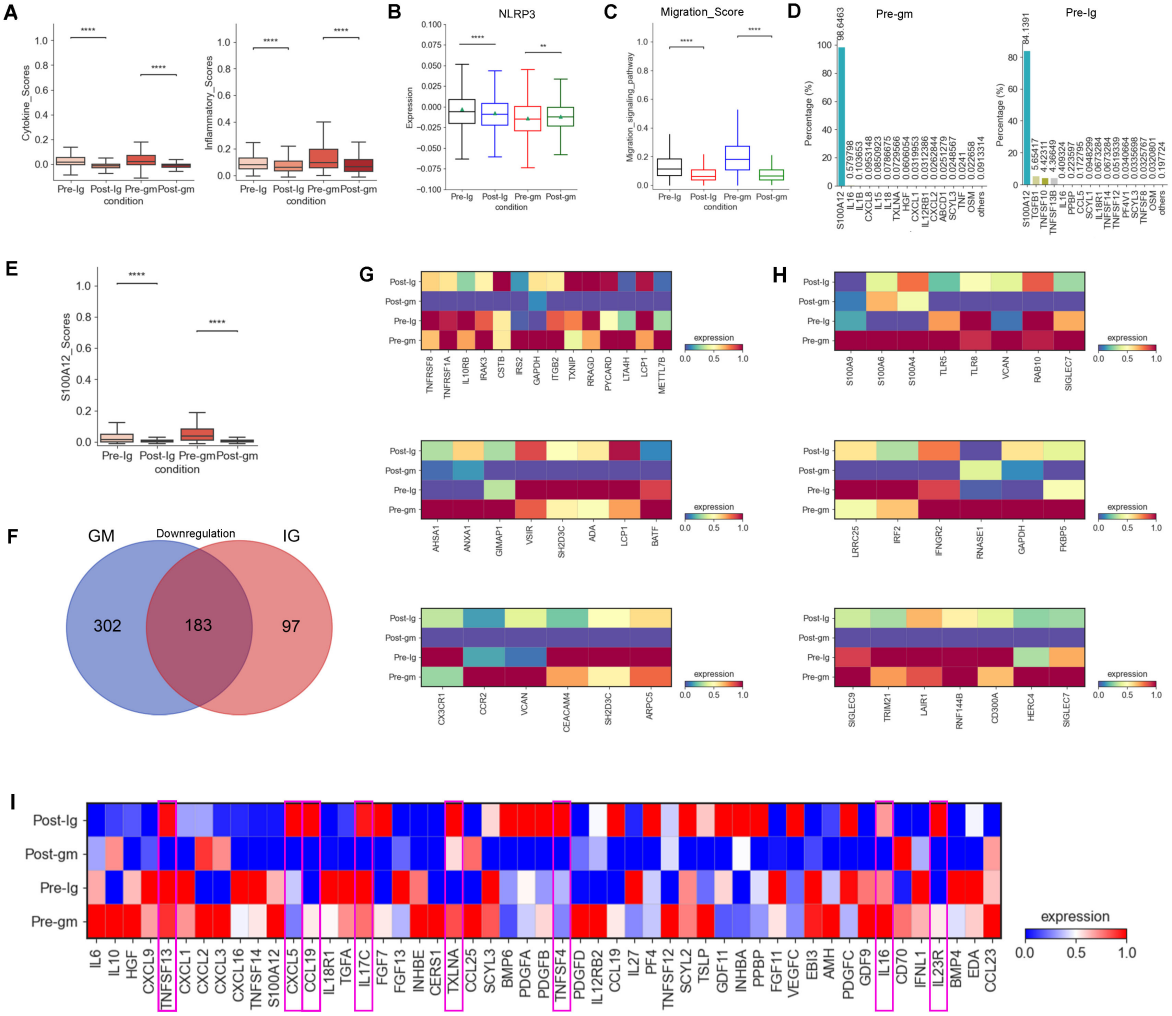
Changes in inflammatory response and cytokines after treatment with different therapies. **(A)** Box plots of the cytokine score (left panel) and inflammatory score (right panel) in PBMCs from Pre-Ig, Post-Ig, Pre-gm, and Post-gm samples. **(B)** Box plot showed the expression level of NLRP3 in monocytes from each group. **(C)** Box plot portrayed the expression level of genes associated with migration in monocytes from each group. **(D)** The bar graph displayed proportion of different cytokines in monocytes from Pre-gm (left panel) and Pre-Ig (right panel) conditions. **(E)** Box plot showed the expression level of S100A12 in monocytes from each group. **(F)** The Venn diagram showed DEGs of downregulation in different groups before- and after- therapy across monocytes. Blue circle showed DEGs in patients with IVIG plus methylprednisolone therapy. Red circle indicated DEGs in patients with IVIG only therapy. **(G)** Heatmap portrayed the expression of DEGs related to cytokine/chemokines, inflammatory response activation, and T cell activation/differentiation. **(H)** Heatmap displayed the expression of DEGs associated with S100A-TLR signaling pathway and interferon-related response. **(I)** Heatmap portrayed the expression of individual cytokines in monocytes from Pre-Ig, Post-Ig, Pre-gm, and Post-gm conditions. **p ≤ 0.01; ****p ≤ 0.0001.

In our prior research, we confirmed that monocytes are the primary inflammatory cells in acute KD (27). Consequently, we calculated inflammatory response and cytokine scores in monocytes, noting a notable reduction in these scores across various treatment groups. The decrease was particularly pronounced with IVIG plus methylprednisolone therapy (**Figure 2A**). Moreover, monocytes displayed reduced levels of NLRP3 inflammasome, a crucial component of the inflammatory cascade, following both therapeutic interventions (**Figure 2B**). Genes associated with the migration-signaling pathway of inflammatory cells showed similar decreases in monocytes following both treatment regimens (**Figure 2C**). These findings indicate that the treatment of KD alleviates the disease by suppressing factors linked to the inflammatory response in monocytes

We next explored the expression of pro-inflammatory cytokines involved in the inflammatory response in KD. *S100A12*, a granulocyte-derived agonist of both RAGE and TLR4 (28), was highly expressed by monocytes before the therapy (**Figure 2D**). Both IVIG monotherapy and combination therapy with methylprednisolone were effective in suppressing *S100A12* expression (**Figure 2E**), suggesting that targeting *S100A12* inhibitors in monocytes could be a promising therapeutic strategy for KD.

To understand the different intrinsic mechanisms of IVIG monotherapy and IVIG plus methylprednisolone therapy, we analyzed DEGs by comparing monocytes from the pre-treatment group and the post-treatment group under both treatment strategies. Notably, 302 DEGs were specifically downregulated in the IVIG plus methylprednisolone therapy group (**Figure 2F**). Grouping these DEGs into functional modules revealed their primary roles in “cytokine/inflammatory response activation”, “S100A-TLR signaling pathway”, “interferon-related response”, “T cell activation/differentiation” and “chemokines” pathways (**Figures 2G, H**). These results suggested that IVIG combined with methylprednisolone therapy could suppress the inflammatory response through a broader spectrum of intrinsic mechanisms relative to IVIG alone.

We also compared cytokine changes pre- and post-treatment in both IVIG monotherapy and combination therapy with methylprednisolone. We discovered that IVIG plus methylprednisolone therapy significantly reduced the expression levels of various pro-inflammatory cytokines in KD patients, including TNFSF13, CXCL5, CCL19, IL17C, TXLNA, TNFSF4, IL16 and IL23R, a phenomenon not observed with IVIG monotherapy (**Figure 2I**). These findings suggested that IVIG plus methylprednisolone has a more pronounced inhibitory effect on cytokines. Consequently, these cytokines may represent therapeutic targets for methylprednisolone’s anti-inflammatory effects. Therefore, methylprednisolone therapy may be more effective in cases where these cytokines are significantly overexpressed in KD patients.

### 3.4 IVIG plus methylprednisolone increased the cytotoxicity of NK cells by regulating the balance of receptors

**Figure 3:**
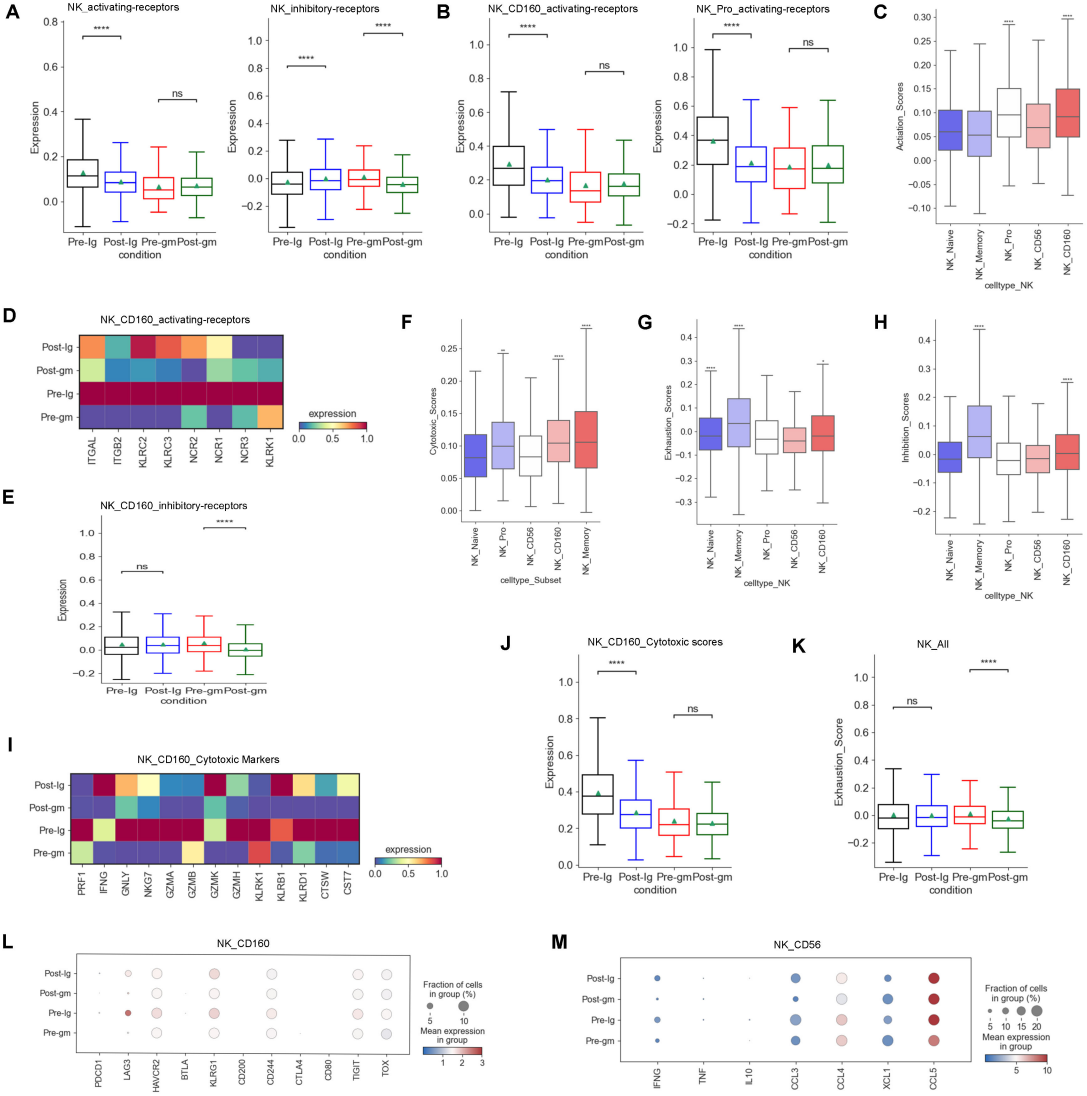
Characteristics of NK cells cytotoxicity and receptors after different therapies. **(A)** Box plots showing the scores of activating receptors and inhibitory receptors in NK cells from each group respectively. **(B)** Box plots portrayed the scores of activating receptors in NK_CD160 and NK_Pro subclusters from each group respectively. **(C)** The expression of activating receptors in all NK cell subclusters. **(D)** Heatmap displayed the activating receptors in NK_CD160 subcluster from each group. **(E)** Box plots showed the scores of the inhibitory receptors in NK_CD160 subcluster from each group. **(F)** The expression of cytotoxic scores in all NK cell subclusters. **(G)** Expression activity of exhaustion genes in all NK cell subclusters. **(H)** The expression of inhibitory receptors in all NK cell subclusters. **(I)** Heatmap showed the expression of individual cytotoxic markers in NK_CD160 subtype from each group. **(J)** Box plots portrayed normalized average expression of the cytotoxic markers in NK_CD160 subtype from each group. **(K)** Box plots displayed the expression of the exhaustion genes in all NK cells derived from each group. **(L)** Expression activity of individual exhaustion genes in NK_CD160 subtype derived from Pre-Ig, Pre-gm, Post-Ig, and Post-gm conditions. **(M)** Dot plots displayed the expression of individual cytokines in NK_CD56 subtype from each group. ns: p > 0.05; *p ≤ 0.05; **p ≤ 0.01; ****p ≤ 0.0001.

We analyzed multiple functions of NK cells in the present study. First, we investigated the expression of activating and inhibitory receptors in NK cells, as their cytotoxic effects rely on a delicate balance between these receptors (29). Our data showed that the activating-receptor score was increased by IVIG plus methylprednisolone therapy, whereas it was decreased by IVIG-alone therapy (**Figure 3A**). However, the inhibitory receptor scores displayed an inverse pattern compared to the activating receptor scores between the two different treatment groups (**Figure 3A**). These results suggested that IVIG plus methylprednisolone may restore the activating receptor expression, thereby activating the function of NK cells. Further analysis of NK cell subtypes revealed that the activating receptors scores of NK_Pro and NK_CD160 subtypes were higher than those of other subtypes (**Figure 3C**). IVIG plus methylprednisolone therapy increased the levels of the activating receptors for the NK_Pro and NK_CD160 subtypes when compared to the IVIG monotherapy (**Figure 3B**). Further analysis showed that the major upregulated activating receptors in NK_CD160 cells of the IVIG plus methylprednisolone therapy group were ITGAL, ITGB2, KLRC2, KLRC3, and NCR1(**Figure 3D**). Additionally, the inhibitory receptors of the NK_CD160 subtype were reduced in the IVIG plus methylprednisolone treatment group, whereas they were elevated in the IVIG-only group (**Figure 3E**). Collectively, these results indicated that IVIG plus methylprednisolone treatment activated the NK_CD160 cell subtype.

Second, we examined the expression of cytotoxic genes in NK cells. Our research revealed that among various subtypes of NK cells, the NK_Memory, NK_Pro, and NK_CD160 subtypes exhibited the highest expression of cytotoxicity genes (**Figure 3F**). We observed that the cytotoxicity score of the NK_CD160 subtype was dramatically decreased after treatment with IVIG alone, however, it appeared to be increased after IVIG plus methylprednisolone treatment (**Figure 3J**). Further studies showed that *GNLY* and *NKG7* were dramatically down-regulated following IVIG monotherapy, while displaying an up-regulation trend after IVIG plus methylprednisolone therapy in the NK_CD160 subtype (**Figure 3I**). Thus, *GNLY* and *NKG7* within the NK_CD160 subtype may be important loci affected by IVIG plus methylprednisolone treatment. We also investigated the exhaustion levels of NK cells. Overall, the exhaustion level of NK cells decreased after treatment with IVIG plus methylprednisolone (**Figure 3K**). These results suggest that IVIG plus methylprednisolone can reduce the exhaustion level of NK cells to maintain their cytotoxic function.

In addition to the cytotoxic function, the secretion of cytokines or chemokines is also a crucial aspect of NK cell function. Our previous study found that NK cells in KD patients produce higher levels of cytokines (CCL3, CCL4, CCL5 and XCL1) (27). Hence, we compared the changes in cytokines caused by different treatment regimens. Our findings showed that a more noticeable down-regulation trend in cytokines following IVIG plus methylprednisolone treatment, particularly in IFNG, CCL3 and CCL4 (**Figure 3M**)

### 3.5 IVIG plus methylprednisolone inhibited B cell activation and increased type I interferon expression

**Figure 4:**
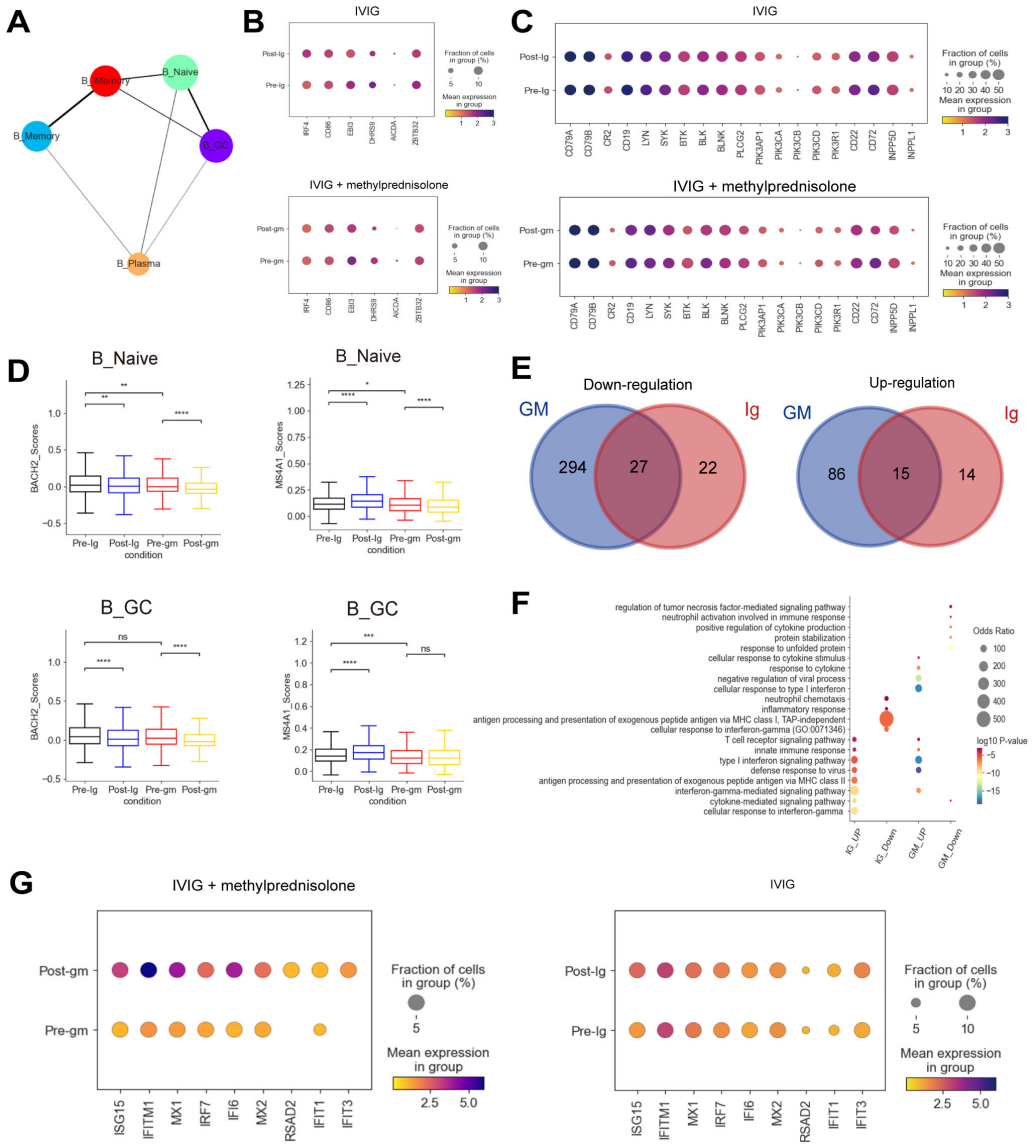
Immune features of B cells in patients with KD using different therapies. **(A)** A graph abstraction of the differential pathway of B cells. **(B)** Dot plots showed the expression of genes associated with the activation of B cells in different groups. **(C)** Dot plots displayed the expression of BCR-related genes in different groups. **(D)** Box plots illustrating the expression of activation-associated genes in the B_Naive and B_GC subclusters. **(E)** The Venn diagrams portrayed the differential expressed genes (DEGs) between KD patients who received IVIG or IVIG plus methylprednisolone before and after therapy in B cells. Blue circle represented DEGs in patients with IVIG plus methylprednisolone therapy. Red circle indicated DEGs in patients with IVIG only therapy. DEGs refer to genes with Benjamini-Hochberg adjusted p value (two-sided unpaired Mann-Whitney U-test) ≤ 0.01 and average log2 fold change ≥ 1 in both FJ/NJ and SJ/NJ comparisons. **(F)** GO enrichment analysis of DEGs identified by comparing before and after therapy with IVIG (IG) or IVIG plus methylprednisolone (GM) in B cells. **(G)** The expression of interferon related genes in the patients before and after with IVIG plus methylprednisolone (left panel) or IVIG therapy (right panel). ns: p > 0.05; *p ≤ 0.05; **p ≤ 0.01; ***p ≤ 0.001; ****p ≤ 0.0001.

The dynamic of plasma B cells differed between the two treatment groups, which increased following IVIG treatment but decreased after IVIG plus methylprednisolone treatment (**Supplementary Figure S3A**). To further investigate the origin of plasma cells in our study, we performed PAGA analysis, which showed that plasma B cells were differentiated from B_Naive, B_GC, and B_Memory (**Figure 4A**). An analysis of transcriptional factors (TFs) related to memory B cell activation showed that the expression of *IRF4* and *AICDA* were elevated in KD patients treated with IVIG, but not in patients treated with IVIG plus methylprednisolone (**Figure 4B**). *IRF4* and *AICDA* play key roles in promoting the differentiation of plasma B cells (30). Therefore, plasma B cells may be differentiated from memory B cells. *MS4A1* and *BACH2*, associated with early B cell activation stages, were significantly reduced in naïve B cells and germinal center (GC) B cells following IVIG plus methylprednisolone therapy (**Figure 4D**). These results suggest that IVIG plus methylprednisolone may impair the proliferation of plasma B cells by hindering early and memory B cell activation. We also investigated the expression of B cell receptor (BCR)-related genes and found that the expression of *CR2* and *CD19*, which encode the two components of the B cell co-receptor complex that enhances BCR-mediated signaling (31), was decreased in response to IVIG plus methylprednisolone treatment (**Figure 4C**). We also noticed a drop in *BTK* expression, which encodes important tyrosine kinases that are downstream of the BCR complex. The expression of the key B cell effector gene *PLCG2* and *PIK3CD* was also reduced in response to IVIG plus methylprednisolone (**Figure 4C**). These results suggest methylprednisolone strongly suppressed the expression associated with BCR signaling pathway, consistent with previous studies (32). B cells can influence the activation of inflammatory signaling pathways through interactions with other immune cells, thereby modulating the intensity and duration of inflammatory responses. Autoimmune diseases like rheumatoid arthritis and systemic lupus erythematosus have been linked to abnormal B cell activation (33).

Next, we plotted Venn diagrams to portray the shared and unique DEGs between KD patients who received IVIG or IVIG plus methylprednisolone treatment in B cells (**Figure 4E**). Interestingly, most of the upregulated (86/101, 85.1%) and downregulated (294/321, 91.6%) DEGs in the IVIG plus methylprednisolone treatment group were not shared with the IVIG treatment group, which suggested that methylprednisolone treatment might cause unique transcriptomic changes in B cells of KD patients. Then, GO terms enrichment analysis on these DEGs provided insights into B cell functional dynamics (**Figure 4F**). Genes in the “antigen processing and presentation of exogenous peptide antigen via MHC class II” pathway were enriched after IVIG treatment, implying that antigen presentation and conversion may have been initiated. For patients treated with IVIG plus methylprednisolone, the “cellular response to type I interferon” pathway was specifically upregulated, and the representative genes include *ISG15*, *IFITM1*, and *MX1* (**Figures 4F–G**). *ISG15*, a key member of interferon-stimulated genes (ISGs), regulates immune response against pathogen invasion (34). *IFITM1* is crucial for antiviral activities, mainly inhibiting the fusion of enveloped RNA viruses with cellular membranes (35). *MX1* encodes mycovirus resistance protein A (MxA), an interferon-induced antiviral guanosine triphosphatase that inhibits the replication of RNA and DNA viruses (36). We also performed a GO analysis of DEGs in CD4^+^ T cells and CD8+ T cells. Similar to B cells, there was an upregulation of pathways and genes related to interferon and interferon-induced apoptosis after treatment with IVIG plus methylprednisolone (**Supplementary Figures S5C-E**). These results indicated the importance of the IFN response in the anti-inflammatory process of KD.

### 3.6 IVIG plus methylprednisolone increased the expression of CD4^+^ Treg cells

**Figure 5:**
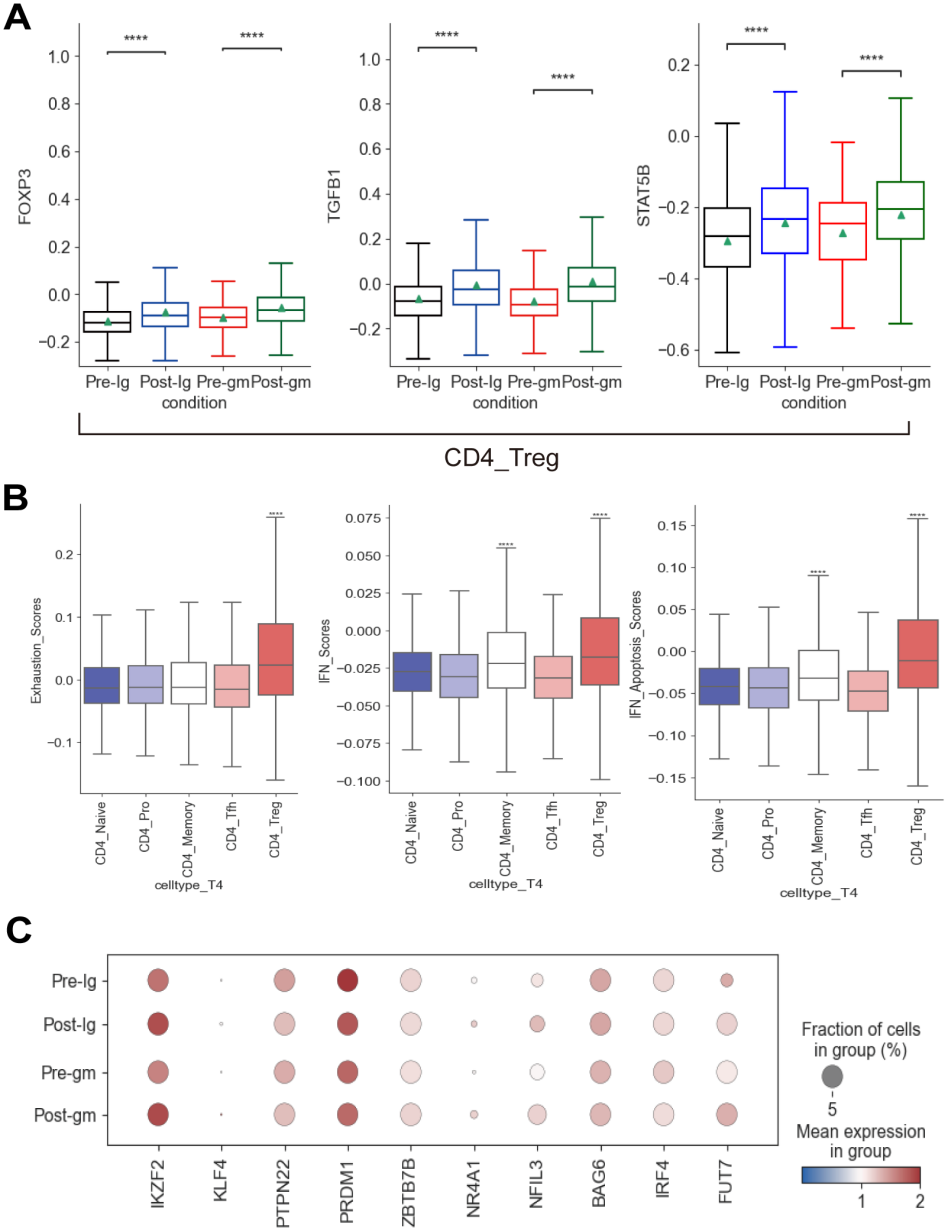
Immune features of CD4^+^ T cells in KD patients after treatment. **(A)** Box plots showing the expression of transcription factors associated with CD4_Treg subcluster in different groups. **(B)** Box plots showing the exhaustion scores, IFN scores, and IFN-related apoptosis scores of CD4^+^ T subclusters. **(C)** Dot plots displaying the expression of exhausted markers in CD4_Treg cells. ****p ≤ 0.0001.

The proportion of CD4_Treg and CD4_Memory subtypes increased in both post-treatment groups (**Supplementary Figure S3B**). Next, we explored the TFs associated with the development and activation of CD4_Treg cells. *FOXP3*, *TGFB1*, and *STAT5B* were significantly elevated following IVIG plus methylprednisolone therapy and IVIG monotherapy (**Figure 5A**). *FOXP3* is a lineage-specific TF for CD4_Treg cells that participate in their development and regulation. *TGFB* plays a key role in the induction of *FOXP3* as well as the development and function of CD4_Treg cells (37). The increase in these TFs indicated that CD4_Treg cells were significantly activated after treatment. CD4_Treg cells are indispensable immune cells that regulate the immune responses and suppress autoimmune reactions. They can exert immunosuppressive effects by secreting inhibitory cytokines (e.g. IL4, IL10, etc.), or directly killing CD8^+^ effector T cells with granzyme and perforin. Hence, the inflammatory response was inhibited by an increase in CD4_Treg cell expression following both IVIG plus methylprednisolone and IVIG monotherapy. Furthermore, we evaluated the factors associated with CD4^+^ T cell exhaustion (38). Among all CD4^+^ T subtypes, the exhaustion score of CD4_Treg cells exhibited a significant increase (**Figure 5B**). The expression of *IKZF2*, *KLF4*, *NR4A1*, *FUT7*, and *NFIL3* in CD4_Treg was noticeably raised after treatments (**Figure 5C**). These results suggested that CD4_Treg cells may increase the expression of CD4^+^ T cell exhaustion by up-regulating *IKZF2*, *KLF4*, *NR4A1*, *FUT7*, and *NFIL3*, thereby reducing cytokine production.

### 3.7 Alteration in CD8^+^ T cell subset transcriptomes after IVIG or IVIG plus methylprednisolone therapy

**Figure 6:**
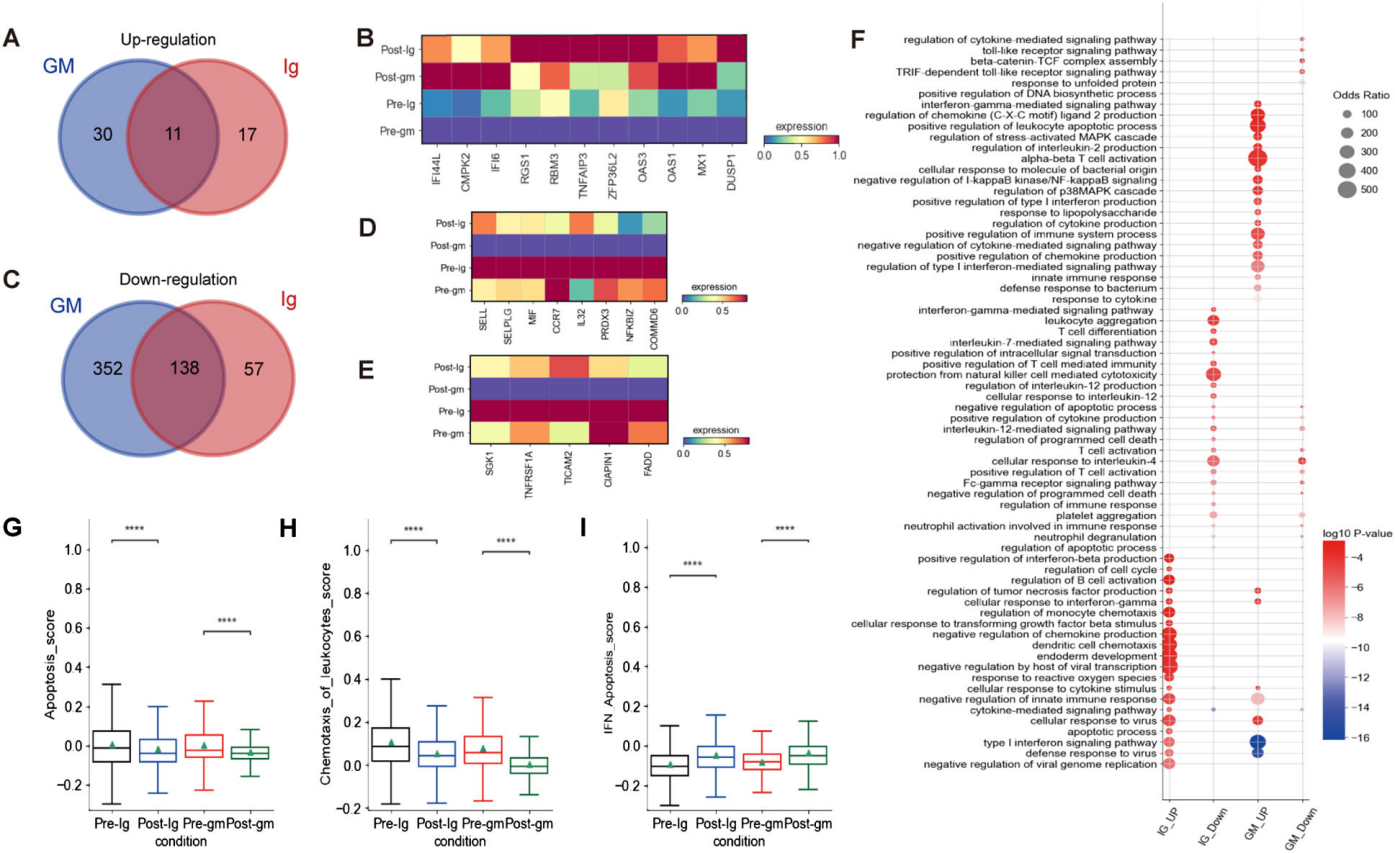
The inflammatory pathways of CD8+ T cells were blocked after treatment. **(A)** The Venn diagram portrayed DEGs of upregulation between KD patients who received IVIG or IVIG plus methylprednisolone before and after therapy in CD8+T cells. **(B)** Heatmap illustrating the expression of 11 shared DEGs in different groups based on the Venn diagram Venn diagram A.**(C)** The Venn diagram showed DEGs of downregulation in different groups before and after therapy across CD8+T cells. Blue circle showed DEGs in patients with IVIG plus methylprednisolone therapy. Red circle indicated DEGs in patients with IVIG therapy. **(D)** Heatmap showed the gene expression related to leukocyte rolling, chemotaxis, and the NF-κB signaling pathway in different groups of CD8+ T cells. **(E)** Heatmap displayed gene expression associated with apoptosis across different groups of CD8+ T cells. **(F)** GO enrichment analysis of DEGs identified by comparing before and after therapy with IVIG (IG) or IVIG plus methylprednisolone (GM) in CD8+T cells. **(G)** Box plots displayed the expression of genes related to apoptosis in different groups. **(H)** Box plots showed the expression of genes associated with chemotaxis of leukocytes in different groups. **(I)** Box plots indicated the expression of genes related to IFN-apoptosis in different group. ****p ≤ 0.0001.

We characterized DEGs in CD8^+^ T cells before and after treatment in two different treatment groups. We identified 11 shared DEGs that were elevated in both groups, including *IFI44L*, *CMPK2*, *IFI6*, *RGS1*, *RBM3*, *TNFAIP3*, *ZFP36L2*, *OAS3*, *OAS1*, *MX1*, and *DUSP1* (**Figures 6A, B**). Notably, *ZFP36L2* and *DUSP1* are known to facilitate the activation of the MAPK signaling pathway in inflammatory responses. We also observed 138 shared DEGs that were down-regulated in both treatment groups. These include genes that promote leukocyte rolling and chemotaxis (e.g. *SELL*, *SELPLG*, *MIF*, *CCR7*), as well as genes that modulate the NF-κB signaling pathway (e.g. *IL32*, *PRDX3*, *NFKBIZ*, *COMMD6*) (**Figures 6C, D**). In the IVIG plus methylprednisolone treatment group, we additionally discovered significantly down-regulated DEGs involved in apoptosis, including *SGK1*, *TNFRSF1A*, *TICAM*, *CIAPIN1*, and *FADD* (**Figure 6E**)Moreover, both apoptosis scores and chemotaxis of leukocyte scores exhibited a greater reduction after IVIG plus methylprednisolone therapy compared to IVIG monotherapy (**Figures 6G, H**).

To further investigate the function of CD8^+^ T cells, we conducted GO enrichment analysis on the up-regulated and down-regulated DEGs (**Figure 6F**). GO analysis revealed enrichment of pathways after IVIG plus methylprednisolone treatment, including “regulation of stress-activated MAPK cascade”, “regulation of p38MAPK cascade”, and “negative regulation of I-kappaB kinase/NF-kB signaling” (**Figure 5F**). These findings suggested that NF-kB and MAPK signaling pathways were involved in the mechanisms of KD. Further research is needed to confirm their regulatory effects on immune cells in KD patients and their connection to the inflammatory response mediated by these pathways.

## 4 Discussion

In this study, we investigated the mechanisms underlying different therapies for KD through the analysis of single-cell transcriptomes. Our findings revealed distinct cellular features and intrinsic mechanisms associated with IVIG plus methylprednisolone treatment compared to IVIG monotherapy. Our results revealed that IVIG plus methylprednisolone showed a stronger inhibitory effect on inflammation during the acute stage of KD. Specifically, it increased the number of lymphocytes (such as CD4^+^T and CD8^+^T cells) and decreased the number of inflammatory cells (such as monocytes). Moreover, IVIG plus methylprednisolone therapy inhibited B cell activation and upregulated the expression of interferon-related genes, especially those of the type I interferon category, in CD4^+^T cells, CD8^+^T cells, and B cells. This therapy also uniquely enhanced NK cell cytotoxicity by regulating receptor homeostasis and significantly reduced the expression level of pro-inflammatory cytokines (e.g. *CXCL5*, *CCL19*, *IL17C*, et, al.).

Monocytes, which play a crucial role in inflammation, were identified as one of the primary inflammatory cells during the acute phase of KD and emerged as the major source of cytokine storms, especially in patients with CAAs (27). In the present study, we found that the combination of IVIG and methylprednisolone more effectively inhibited multiple monocyte-driven inflammatory pathways than IVIG monotherapy. These findings suggest that the combination of IVIG and methylprednisolone therapy may be beneficial for patients with monocyte-driven hyperinflammatory response in KD. *S100A12*, also known as EN-RAGE (the newly identified extracellular RAGE binding protein), acts as a receptor triggering downstream signaling pathway, notably the NF-κB pathway, which can lead to endothelial damage and promotes KD (39, 40). In the present study, we observed a dramatic elevation of *S100A12* expression during the acute phase of KD, followed by a significant drop after therapy. Therefore, it is reasonable to consider targeted inhibition of *S100A12* as a novel treatment approach for KD. Additionally, our findings implied that IVIG plus methylprednisolone or IVIG monotherapy can also effectively lower NLRP3 expression. In a mouse model of KD induced by Lactobacillus casei cell wall extract (LCWE), the NLRP3 inflammasome was activated in the coronary endothelium, leading to a significant increase in caspase1 activity and IL-1β production. Conversely, inhibiting the NLRP3 inflammasome expression reversed the extent of coronary endothelial damage (41, 42). Thus, blocking NLRP3 inflammasome could be also a significant targeted treatment for KD.

To further understand the underlying mechanism of IVIG plus methylprednisolone therapy, we mapped the DEGs in monocytes before and after therapy. According to our data, IVIG plus methylprednisolone downregulated DEGs in multiple functional modules, including T cell activation, cytokines, inflammatory response, and S100A-TLR signaling pathway, indicating a more potent anti-inflammatory effect. This suggests that IVIG plus methylprednisolone therapy involves multiple ways of the inflammatory response process, resulting in a more pronounced anti-inflammatory effect. Focusing on cytokines, our research discovered that IVIG treatment alone failed to significantly reduce the expression of TNFSF13, CXCL5, CCL19, IL17C, TXLNA, TNFSF4, IL16, and IL23R cytokines. However, these cytokines could be significantly reduced by adding methylprednisolone to the regimen, suggesting that methylprednisolone may target these specific cytokines.

IVIG plus methylprednisolone effectively dampened the inflammatory response by balancing receptors and boosting NK cell cytotoxicity. NK cells are key components of the innate immune system, serving as a vital bridge to adaptive immunity. They exert significant influence on immune responses through their cytotoxic actions and cytokine secretion and are implicated in the pathogenesis of many immune-mediated diseases such as ankylosing spondylitis, Behcet’s disease, multiple sclerosis, rheumatoid arthritis, psoriasis, systemic lupus erythematosus, and type 1 diabetes (43–46). Previous understanding indicated that NK cell activity is governed by a delicate balance of various activating and inhibitory receptors on their surface (29). NK cells constantly surveil neighboring cells for abnormalities and eliminate virus-infected and malignant cells. IVIG has been reported to inhibit NK cell cytotoxicity in other studies (47, 48), our findings are consistent with previous reports. Differently, the combination of IVIG and methylprednisolone enhanced NK cell cytotoxicity, which resulted in the rapid removal of aberrant inflammatory cells and pro-inflammatory cytokines to curb excessive inflammatory responses. These findings suggest that IVIG combined with methylprednisolone could be an effective treatment for patients with impaired NK cell function. Moreover, the combination therapy of IVIG and methylprednisolone prominently increased the expression of interferon-related genes in B cells, CD4^+^ T cells, and CD8^+^ T cells, particularly enhancing the type I interferon response. Previous studies have highlighted the pivotal role of the interferon response in various inflammatory and autoimmune conditions, though its specific mechanisms in KD remain elusive. Our study observed an elevation of apoptosis-related genes following treatment, elucidating a close relationship between interferon-related genes and apoptotic functions, with many IFN-stimulated genes exhibiting apoptotic properties (49). Further investigation revealed an upsurge in the expression of *LGALS9*, *OAS1*, and *EIF2AK2* genes following KD treatment, each playing distinctive roles in inducing apoptosis and antiviral effects (35, 36, 50–52). These results suggest that the interferon response may mitigate inflammatory responses by inducing apoptosis and exerting antiviral effects, thereby suggesting the potential of interferon therapy, particularly type I interferon therapy, as a novel therapeutic avenue for KD.

This study has some limitations. Firstly, although it is already the largest sample reported by far on the basis of the current conditions, the sample size is small which raises concerns about the robustness and generalizability of the findings. Secondly, the age and disease severity were potential confounding variables in the present study. Age, gender and disease severity should be match as much as possible in further studies. Finally, our findings are preliminary and require further systematic experimental validation and clinical evaluation of patients based on the single-cell sequencing results.

In conclusion, we provided a comprehensive analysis of the intrinsic mechanisms underlying the effects of IVIG plus methylprednisolone in KD at a single-cell resolution. We observed that IVIG plus methylprednisolone suppressed more monocyte-mediated inflammatory responses than IVIG alone. This combination also enhances NK cell cytotoxicity and inhibits B cell activation, effects not observed with IVIG alone. These findings may contribute to the development of new targeted therapies for KD.

## Data Availability

All data and materials are publicly available at the China National Center for Bioinformation and can be accessed via the following links: https://ngdc.cncb.ac.cn/omix/release/OMIX004848 and https://ngdc.cncb.ac.cn/omix/release/OMIX007642.
