# Supplementary material for: Single-cell RNA sequencing uncovers molecular mechanisms of intravenous immunoglobulin plus methylprednisolone in Kawasaki disease: attenuated monocyte-driven inflammation and improved NK cell cytotoxicity
Source: Front Immunol. 2024 Oct 25;15:1455925. doi: 10.3389/fimmu.2024.1455925 (PMC11543420; doi:10.3389/fimmu.2024.1455925)
Supplement: Supplementary file 1 [file DataSheet1.docx]

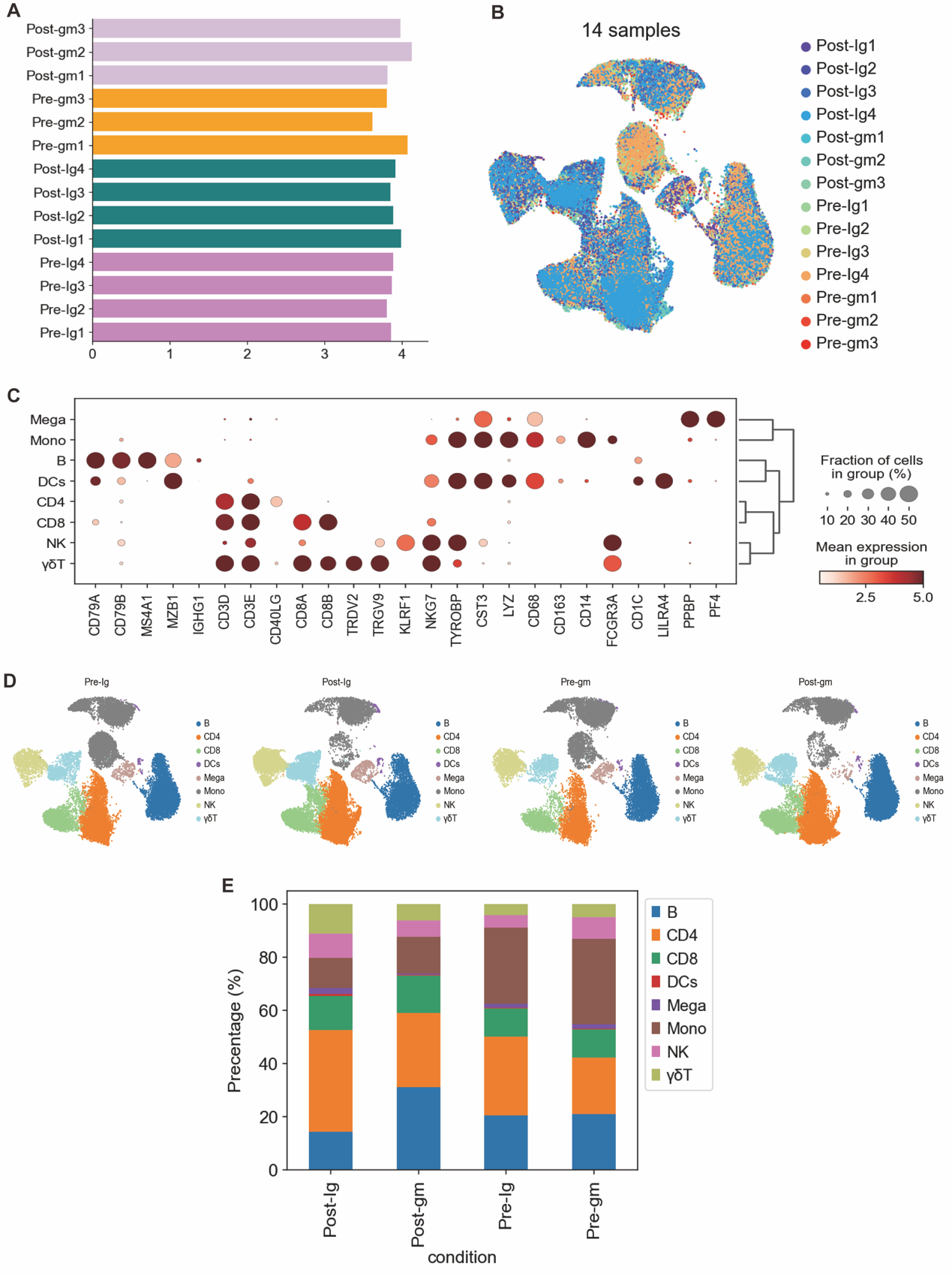
**Figure S1** The fundamental characteristics of immune cells in all samples

**A,** Comparison of the cell amount in each sample. **B,** UMAP plot marking cells from different samples. **C,** Dot plot showing the expression of canonical cell markers for each cell type. **D,** The distribution of all immune cells from different groups. **E,** The percentage of each cell cluster in each group.


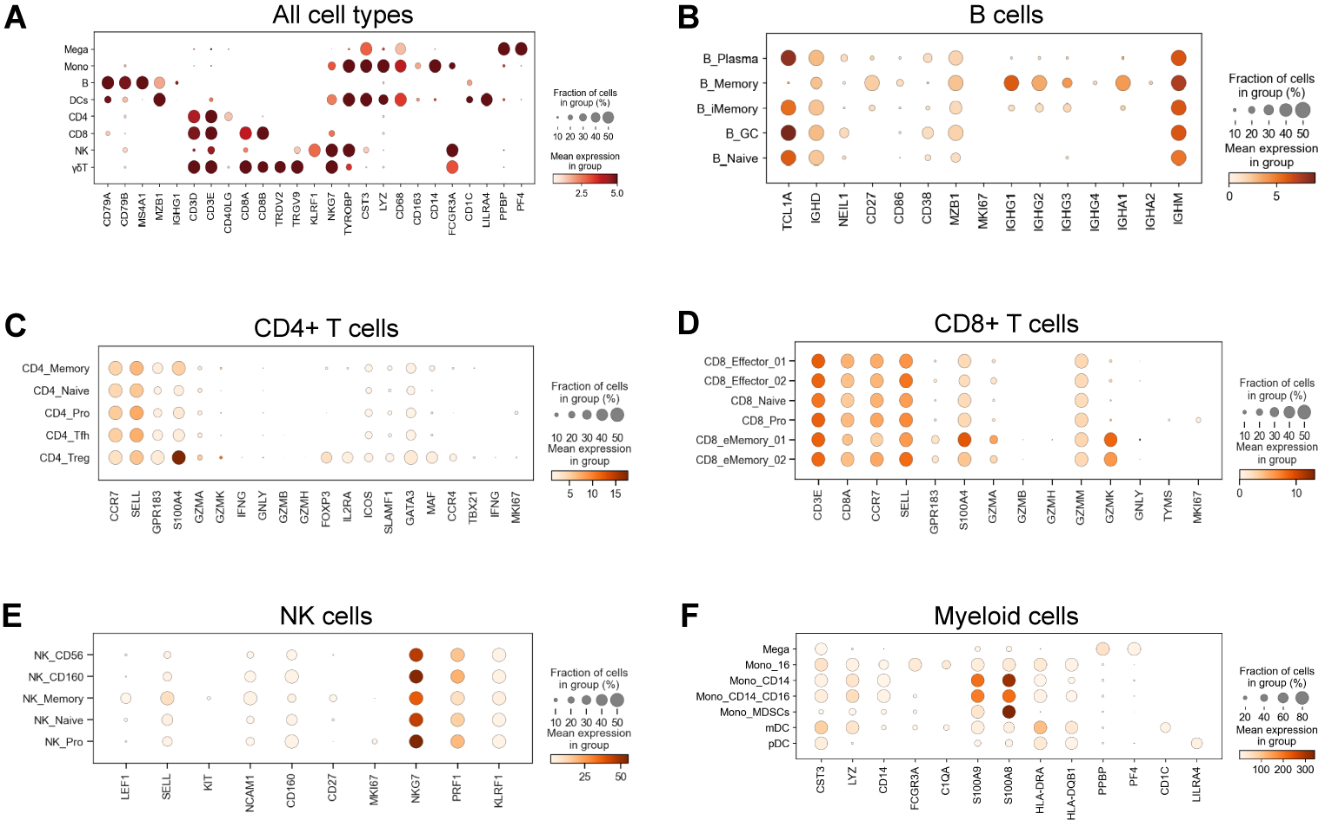


**Figure S2** Cell type identification

**A,** Dot plot showing the expression of canonical cell markers for each major cell type. **B-F,** Dot plot displaying the expression of canonical markers for different cell subclusters.


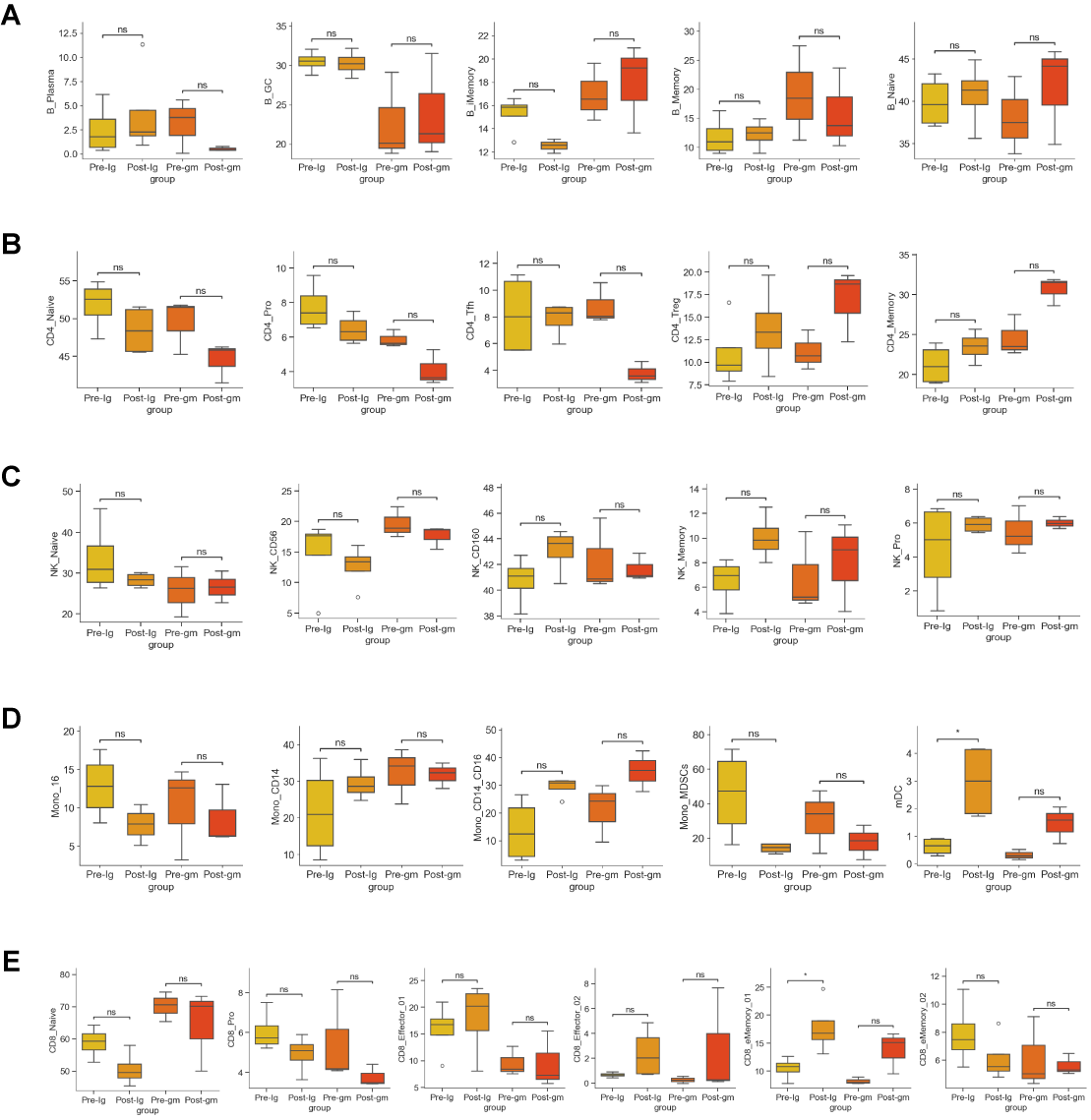


**Figure S3** The proportion of immune cell subclusters

**A,** Box plots comparing the proportion of B cell subclusters across different groups. **B,** Box plots comparing the proportion of CD4+ T cell subclusters across different groups. **C,** Box plots comparing the proportion of NK cell subclusters across different groups. **D,** Box plots comparing the proportion of myeloid cell subclusters across different groups. **E,** Box plots comparing the proportion of CD8+ T cell subclusters across different groups.


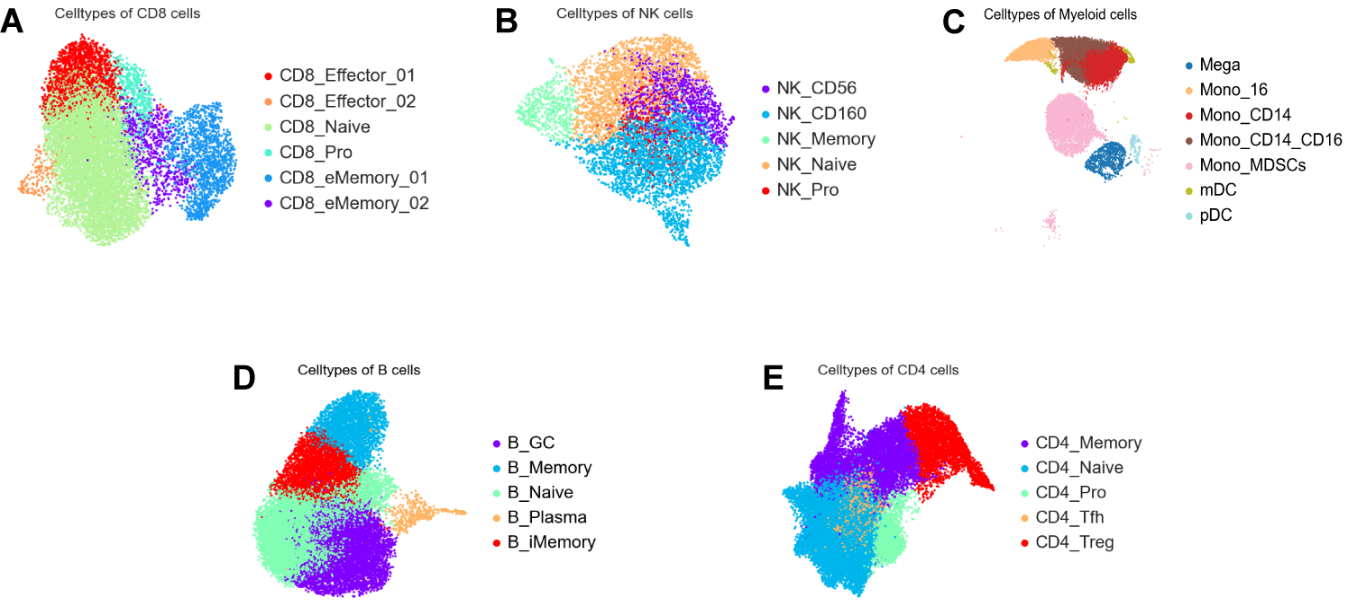


**Figure S4** A two-dimensional visualization classified cell subclusters from major cell clusters

**A,** Identification of peripheral blood CD8+T cell clusters across all samples. **B,** Identification of peripheral blood NK cell clusters across all samples. **C,** Identification of peripheral blood myeloid cell clusters across all samples. **D,** Identification of peripheral blood B cell clusters across all samples. **E,** Identification of peripheral blood CD4+T cell clusters across all samples.

**Figure S5** Additional information about CD4+ T cells

**A,** Dot plots showing the expression of genes associated with cytotoxicity for CD4+T cell subclusters. **B,** The expression of MCH class I and II genes in the patients before- and after- with GM therapy. **C,** Box plots showing the expression of interferon related genes in different groups across CD4_Treg cluster and CD4_Memory cluster. **D,** Box plots showing the expression of interferon induced apoptosis genes in different groups across CD4_Treg cluster and CD4_Memory cluster. **E,** GO enrichment analysis of DEGs identified by comparing before and after therapy with IVIG (IG) or IVIG plus methylprednisolone (GM) in CD4+T cells.

**Table S1** Apoptosis response genes, interferon (IFN) response genes, and IFN-apoptosis genes, related to Fig 5B, Fig 6G, Fig 6I

| **Genes of Apoptosis** | **Genes of IFN response** | **Genes of IFN-apoptosis** |
| --- | --- | --- |
| FAS | ISG15 | TNFSF10 |
| FASLG  TNF, TNFRSF1A, TRADD, NFKB1, MAPK1  TNFSF12, TNFRSF25, TNFRSF10A, TNFSF10  CASP8, CASP3 | IFITM1 | FAS |
| FADD | MX1 | XAF1 |
| TNF | IRF7 | CASP4 |
| TNFRSF1A | IF16 | CASP8 |
| TRADD | MX2 | IP6K2 |
| NFKB1 | RSAD2 | EIF2AK2 |
| MAPK1 | IFIT1 | IRF1 |
| TNFSF12 | IFIT3 | PML |
| TNFRSF25 |  | RNASEL |
| TNFRSF10A |  | LGALS9 |
| TNFSF10 |  | OAS1 |
| CASP8 |  | DAPK1 |
| CASP3 |  | DAPK2 |

**Table S2** Cell exhaustion response genes associated with CD4+ T cells, related to Fig 5B

| **Defined markers of cell exhaustion** |
| --- |
| IKZF2 |
| KLF4 |
| PTPN22 |
| PRDM1 |
| ZBTB7B |
| NR4A1 |
| NFIL3 |
| BAG6 |
| IRF4 |
| FUT7 |

**Table S3** Cell exhaustion response genes and exhaustion markers, related to Fig 3K and Fig 3L

| **Genes of cell exhaustion response** | **Defined markers of cell exhaustion** |
| --- | --- |
| KIAA1671 | PD-1(PDCD1) |
| ABI3 | TIGIT |
| ADAM19 | LAG3 |
| AKNA | CTLA4 |
| ARHGAP9 | HAVCR2 (TIM3) |
| ARL6IP1 | BTLA |
| ARMC7 | CD80 |
| BCL2A1 | CD200 |
| CBX4 | KLRG1 |
| CCL3 | CD244 (2B4) |
| CCL4 |  |
| CCL5 |  |
| CD160 |  |
| CD164 |  |
| CD27 |  |
| CD3E |  |
| CD3G |  |
| CD7 |  |
| CD82 |  |
| CD8A |  |
| CST7 |  |
| CXCR6 |  |
| DAPK2 |  |
| DTX1 |  |
| DUSP2 |  |
| EFHD2 |  |
| EIF4A2 |  |
| FAM189B |  |
| FASLG |  |
| FOXN3 |  |
| FYN |  |
| GIMAP1 |  |
| GIMAP6 |  |
| GIMAP7 |  |
| GLRX |  |
| GNG2 |  |
| GRAMD1A |  |
| GZMA |  |
| GZMB |  |
| GZMK |  |
| HLA-A |  |
| HCST |  |
| HSPA5 |  |
| ID2 |  |
| IFIT3 |  |
| IL21R |  |
| ISG15 |  |
| ITK |  |
| ITPKB |  |
| LAX1 |  |
| LRRK1 |  |
| MBNL1 |  |
| MXD4 |  |
| NR4A2 |  |
| PDCD1 |  |
| PFDN5 |  |
| PLA2G16 |  |
| PLAC8 |  |
| PRDX5 |  |
| PRKCH |  |
| PSMB10 |  |
| PSMB8 |  |
| PSME1 |  |
| PTGER4 |  |
| PTPN18 |  |
| PTPN22 |  |
| RGS1 |  |
| RGS2 |  |
| RGS3 |  |
| RTP4 |  |
| RUNX3 |  |
| SH2D2A |  |
| SHISA5 |  |
| SIPA1 |  |
| SLC3A2 |  |
| STAT1 |  |
| STK17B |  |
| TAP2 |  |
| TAPBP |  |
| TAPBPL |  |
| TNFRSF1B |  |
| TOX |  |
| UCP2 |  |
| VMP1 |  |
| ZBP1 |  |

**Table S4** Inflammatory score and cytokine score, related to Fig 2A

| **Inflammatory scores** | **Cytokine scores** |
| --- | --- |
| ABCA1 | IL2 |
| ABI1 | IL7 |
| ACVR1B | CSF3 |
| ACVR2A | CXCL10 |
| ADGRE1 | CCL2 |
| ADM | CCL3 |
| ADORA2B | TNF |
| ADRM1 | IL6 |
| AHR | CCL7 |
| APLNR | IL1RN |
| AQP9 | CSF1 |
| ATP2A2 | IFNG |
| ATP2B1 | IL2RA |
| ATP2C1 | IL10 |
| AXL | IL18 |
| BDKRB1 | HGF |
| BEST1 | CXCL9 |
| BST2 | CCL27 |
| BTG2 | TGFB1 |
| C3AR1 | IL1B |
| C5AR1 | LTA |
| CALCRL | CSF2 |
| CCL17 | LTB |
| CCL2 | TNFSF13 |
| CCL20 | IL4 |
| CCL22 | CCL12 |
| CCL24 | CXCL8 |
| CCL5 | CXCL11 |
| CCL7 | CCL4 |
| CCR7 | CXCL1 |
| CCRL2 | CXCL2 |
| CD14 | CXCL3 |
| CD40 | CCL3L1 |
| CD48 | CCL8 |
| CD55 | CXCL16 |
| CD69 | IFNA1 |
| CD70 | CCL5 |
| CD82 | CCL11 |
| CDKN1A | IFNA2 |
| CHST2 | CCL20 |
| CLEC5A | CCL4L2 |
| CMKLR1 | OSM |
| CSF1 | TNFSF14 |
| CSF3 | SA100A12 |
| CSF3R | FGF19 |
| CX3CL1 | CXCL5 |
| CXCL10 | CCL19 |
| CXCL11 | IL18R1 |
| CXCL6 | TGFA |
| CXCL8 | IFNB1 |
| CXCL9 | IL8 |
| CXCR6 | IL17C |
| CYBB | TNFSF10 |
| DCBLD2 | FGF7 |
| EBI3 | XCL1 |
| EDN1 | FGF13 |
| EIF2AK2 | LIF |
| EMP3 | TGFB3 |
| EREG | INHBE |
| F3 | CERS1 |
| FFAR2 | TXLNA |
| FPR1 | IFNW1 |
| FZD5 | IL22 |
| GABBR1 | XCL2 |
| GCH1 | CCL25 |
| GNA15 | CCL16 |
| GNAI3 | CD40LG |
| GP1BA | IL20 |
| GPC3 | FASLG |
| GPR132 | TPO |
| GPR183 | SCYL3 |
| HAS2 | PF4V1 |
| HBEGF | TNFSF8 |
| HIF1A | GDF15 |
| HPN | IL1A |
| HRH1 | VEGFA |
| ICAM1 | GDF7 |
| ICAM4 | BMP6 |
| ICOSLG | PDGFA |
| IFITM1 | IL21 |
| IFNAR1 | ABCD-1 |
| IFNGR2 | ABCD-2 |
| IL10 | PDGFB |
| IL10RA | TNFSF4 |
| IL12B | FAM19A1 |
| IL15 | HBEGF |
| IL15RA | PDGFD |
| IL18 | IL12RB2 |
| IL18R1 | GH1 |
| IL18RAP | VEGFB |
| IL1A | MIP3B |
| IL1B | IL27 |
| IL1R1 | PF4 |
| IL2RB | BMP8B |
| IL4R | TNFSF12 |
| IL6 | IL15 |
| IL7R | SCYL2 |
| INHBA | SCYL1 |
| IRAK2 | TSLP |
| IRF1 | GDF11 |
| IRF7 | SDF1B |
| ITGA5 | INHBA |
| ITGB3 | PPBP |
| ITGB8 | FGF11 |
| KCNA3 | IFNG-AS1 |
| KCNJ2 | FGF22 |
| KCNMB2 | VEGFC |
| KIF1B | CCL18 |
| KLF6 | TNFSF11 |
| LAMP3 | IL12A |
| LCK | EBI3 |
| LCP2 | AMH |
| LDLR | IL26 |
| LIF | IL32 |
| LPAR1 | PDGFC |
| LTA | FGF23 |
| LY6E | IGF1 |
| LYN | IL1F11 |
| MARCO | CCL28 |
| MEFV | CLCF1 |
| MEP1A | TNFSF9 |
| MET | BMP3 |
| MMP14 | IL24 |
| MSR1 | GDF10 |
| MXD1 | CXCL6 |
| MYC | GDF9 |
| NAMPT | IL23A |
| NDP | IL16 |
| NFKB1 | CD70 |
| NFKBIA | IL5 |
| NLRP3 | FGF9 |
| NMI | IFNL1 |
| NMUR1 | TSC1 |
| NOD2 | FGF2 |
| NPFFR2 | IL23R |
| OLR1 | IL1G |
| OPRK1 | SPP1 |
| OSM | IL12RB1 |
| OSMR | BMP4 |
| P2RX4 | IL13 |
| P2RX7 | TPAR1 |
| P2RY2 | TGFB2 |
| PCDH7 | FAM19A2 |
| PDE4B | AGIF3 |
| PDPN | EDA |
| PIK3R5 | MIF |
| PLAUR | TNFSF13B |
| PROK2 | BMP7 |
| PSEN1 | FGF18 |
| PTAFR | CCL23 |
| PTGER2 | CCL23 |
| PTGER4 |  |
| PTGIR |  |
| PTPRE |  |
| PVR |  |
| RAF1 |  |
| RASGRP1 |  |
| RELA |  |
| RGS1 |  |
| RGS16 |  |
| RHOG |  |
| RIPK2 |  |
| RNF144B |  |
| ROS1 |  |
| RTP4 |  |
| SCARF1 |  |
| SCN1B |  |
| SELE |  |
| SELENOS |  |
| SELL |  |
| SEMA4D |  |
| SERPINE1 |  |
| SGMS2 |  |
| SLAMF1 |  |
| SLC11A2 |  |
| SLC1A2 |  |
| SLC28A2 |  |
| SLC31A1 |  |
| SLC31A2 |  |
| SLC4A4 |  |
| SLC7A1 |  |
| SLC7A2 |  |
| SPHK1 |  |
| SRI |  |
| STAB1 |  |
| TACR1 |  |
| TACR3 |  |
| TAPBP |  |
| TIMP1 |  |
| TLR1 |  |
| TLR2 |  |
| TLR3 |  |
| TNFAIP6 |  |
| TNFSF14 |  |
| TNFRSF1B |  |
| TNFRSF9 |  |
| TNFSF10 |  |
| TNFSF15 |  |
| TNFSF9 |  |
| TPBG |  |
| VIP |  |

**Table S5** NK cell activating receptors, inhibitory receptors, and cytotoxic genes,

related to Fig 3

| **Genes of activating receptors** | **Genes of inhibitory receptors** | **Genes of cytotoxicity** |
| --- | --- | --- |
| ITGAL | KIR3DL1 | FASLG |
| ITGB2 | KIR3DL2 | ATF2 |
| KIR2DS1 | KIR2DL1 | MAPK14 |
| KIR2DS2 | KIR2DL2 | PTK2B |
| KIR2DS3 | KIR2DL3 | FN1 |
| KIR2DS4 | KIR2DL4 | FOS |
| KIR2DS5 | KLRC1 | GRB2 |
| KLRC2 | KLRD1 | HRAS |
| KLRC3 |  | IFNG |
| NCR2 |  | CXCL8 |
| NCR1 |  | ITGA4 |
| NCR3 |  | ITGAL |
| KLRK1 |  | ITGAM |
|  |  | ITGB1 |
|  |  | ITGB2 |
|  |  | KLRC1 |
|  |  | KLRD1 |
|  |  | PAK1 |
|  |  | PRKCE |
|  |  | MAPK1 |
|  |  | MAPK3 |
|  |  | MAPK11 |
|  |  | MAPK13 |
|  |  | MAP2K1 |
|  |  | MAP2K2 |
|  |  | MAP2K3 |
|  |  | MAPK12 |
|  |  | MAPK14 |
|  |  | PTPN6 |
|  |  | PTPN11 |
|  |  | PXN |
|  |  | RAC1 |
|  |  | RAF1 |
|  |  | ICAM1 |
|  |  | ICAM2 |
|  |  | ICAM3 |
|  |  | LYN |
|  |  | MAP3K1 |
|  |  | SOS1 |
|  |  | SOS2 |
|  |  | VAV1 |
|  |  | VCAM1 |
|  |  | HLA-E |

**Software and algorithms**

| **Software** | **Source** | **Website** |
| --- | --- | --- |
| anadata | pypi | <https://github.com/theislab/anndata> |
| CellRanger v3.x | 10x Genomics | [http://10xgenomics.com](http://10xgenomics.com/) |
| ggplot | bioconductor | [https://ggplot2.tidyverse.org](https://ggplot2.tidyverse.org/) |
| ggpubr | bioconductor | <https://github.com/kassambara/ggpubr> |
| gseapy-0.10.7 | pypi | <https://pypi.org/project/gseapy> |
| harmonypy | pypi | <https://github.com/slowkow/harmonypy> |
| kallistobustools | pypi | <https://github.com/pachterlab/kb_python> |
| scanpy v1.7.2 | bioconda | <https://github.com/theislab/scanpy> |
| scirpy v0.7.0 | bioconda | <https://github.com/icbi-lab/scirpy> |
| scrublet v0.2.3 | pypi | <https://github.com/swolock/scrublet> |
| statannot | pypi | <https://pypi.org/project/statannot> |
